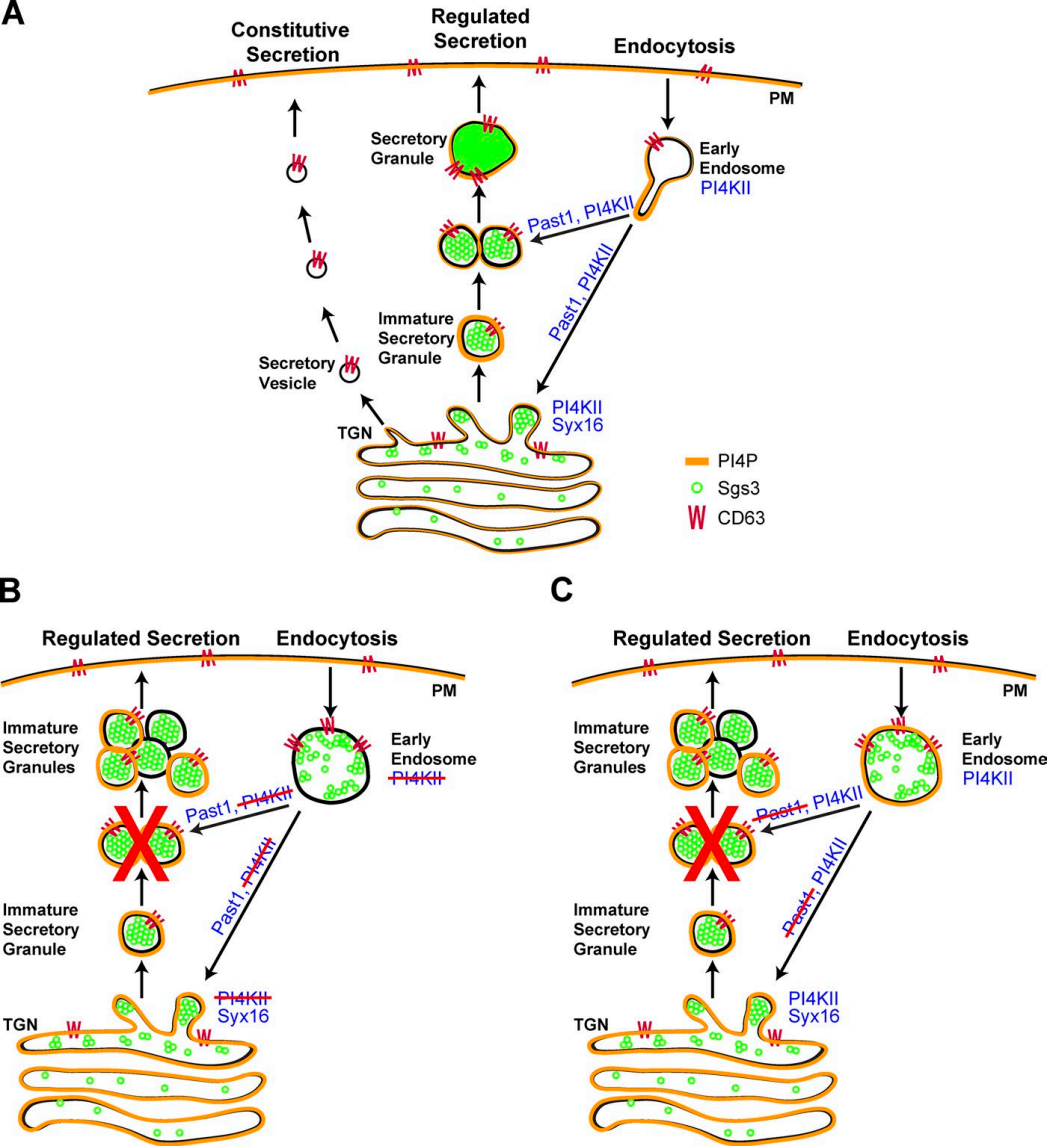# Correction: An early endosome–derived retrograde trafficking pathway promotes secretory granule maturation

**DOI:** 10.1083/jcb.20180801701192021c

**Published:** 2021-01-22

**Authors:** Cheng-I J. Ma, Yitong Yang, Taeah Kim, Chang Hua Chen, Gordon Polevoy, Miluska VIssa, Jason Burgess, Julie A. Brill

Vol. 219, No. 3 | 10.1083/jcb.201808017 | February 11, 2020

In the initial published version of Figure 8 B, "PI4KII" should have been crossed out in all four instances to show that the protein is absent in the *PI4KII* null mutant. The corrected figure is shown here. The authors apologize for any confusion. This error appears only in PDF versions downloaded on or before January 21, 2021.

**Figure:**